# Supplementary material for: Two-stage battery recharge scheduling and vehicle-charger assignment policy for dynamic electric dial-a-ride services
Source: PLoS One. 2021 May 20;16(5):e0251582. doi: 10.1371/journal.pone.0251582 (PMC8136635; doi:10.1371/journal.pone.0251582)
Supplement: S1 Appendix — (DOCX) [file pone.0251582.s001.docx]

## S1 Appendix. Illustrative example for optimal single-vehicle battery recharge (P1).

Consider a single-vehicle battery recharge over a planning horizon from 7:00–22:00. The vehicle is fully charged at the beginning of the day. The planning horizon is divided into a sequence of charging decision epochs with a 30-minute time interval. The probability of the vehicle being in the driving state and the electricity price distribution are shown on the left side of Figure A.1. The expected waiting time to be served at charging stations over the planning horizon is depicted on the right side of Figure A.1. The battery capacity of the vehicle is assumed to be 24 kWh for the first scenario and 48 kWh for the second scenario. Two recharge policies are compared: 1) optimal single-vehicle battery recharge (P1), and 2) the on-need policy, assuming that the vehicle is recharged to 100% of its battery capacity in one epoch whenever its energy level is lower than 20%.

The parameter settings for the illustrative example are shown in Table A.1.


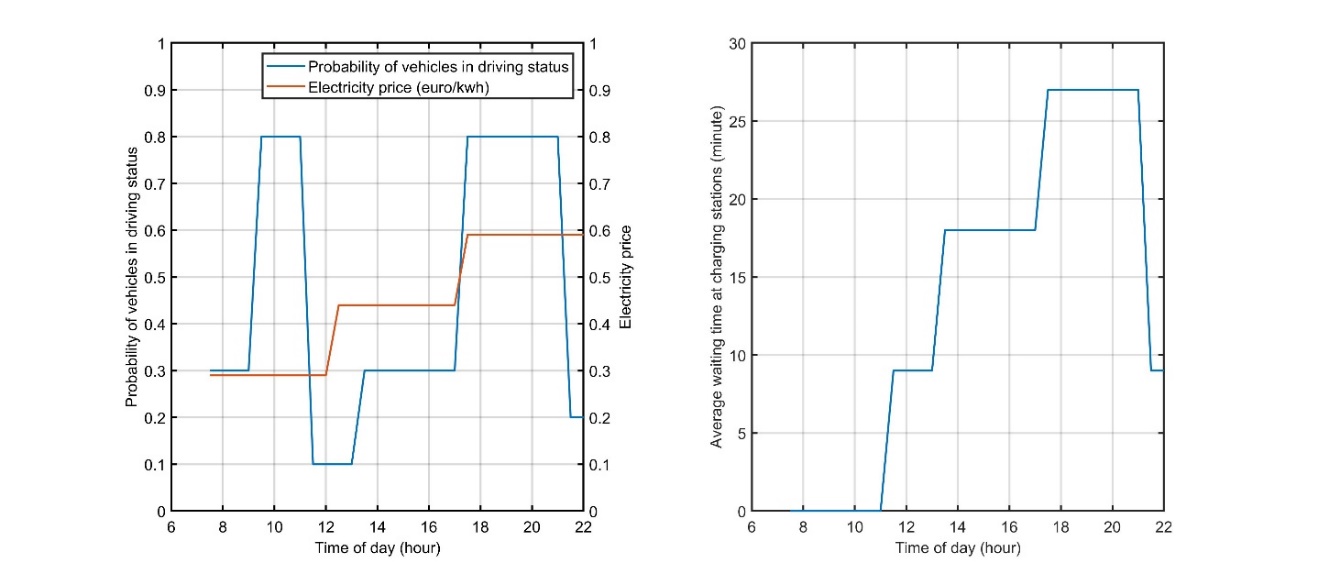


Figure A.1. Probability distributions of the vehicle being in the driving state and the electricity price (euros/kWh) over the charging decision epochs (Left); average waiting time to be served at charging stations over the charging decision epochs (Right).

Table A.1. Parameter settings for the illustrative example.

| Parameter | Value |
| --- | --- |
| $e_{max}$ | 24 kWh (scenario 1) and 48 kWh (scenario 2) |
| $e_{min}$ | $0.1B$ |
| $\varphi$ | 2/3 (kW/min.) |
| $\rho$ | 1/6 (euro/min.) |
| $v$ | 4/6 (km/min.) |
| $\mu$ | 0.2 (kWh/km) |
| $\bar{c}$ | 3 euros |
| $\Delta$ | 30 minutes |
| $T$ | 7:00–22:00 |

Figure A.2 (Left) shows the vehicle recharge profile under the on-need policy. For the case of 24 kWh, the vehicle needs to be recharged twice, from 14:00 to 14:30 and from 19:00 to 19:30, with around 20 kWh charged each time. For the case of 48 kWh, the vehicle goes to recharge from 19:00 to 19:30, with 42 kWh charged. For the optimal battery recharge policy (Figure A.3), the vehicle is recharged from 11:30 to 12:00 (18 kWh charged) and from 17:30 to 18:00 (16 kWh charged) due to a lower driving probability and electricity price, given the 24 kWh battery capacity. If the battery capacity is doubled, the optimal charging time is at 11:30, with 12.7 kWh charged to meet the vehicle’s driving needs until the end of the planning horizon (22:00). The battery level is always no less than $e_{min}.$ Table A.2 compares the total charging costs of the two charging policies. For the optimal battery recharge policy, the total charging operation costs (Eq. (1)) are 31.28 euros (B=24 kWh) and 8.71 euros (B=48 kWh). For the on-need policy, the total costs are 40.3 euros (B=24kWh) and 36.04 euros (B=48kWh). The charging cost savings for the optimal recharge policy are 22.4% (B=24 kWh) and 75.8% (B=48 kWh). The proposed optimal charging plan allows the vehicle to be charged with minimal cost by considering the vehicle’s driving needs, expected waiting times at charging stations, and opportunity cost while not available for service.


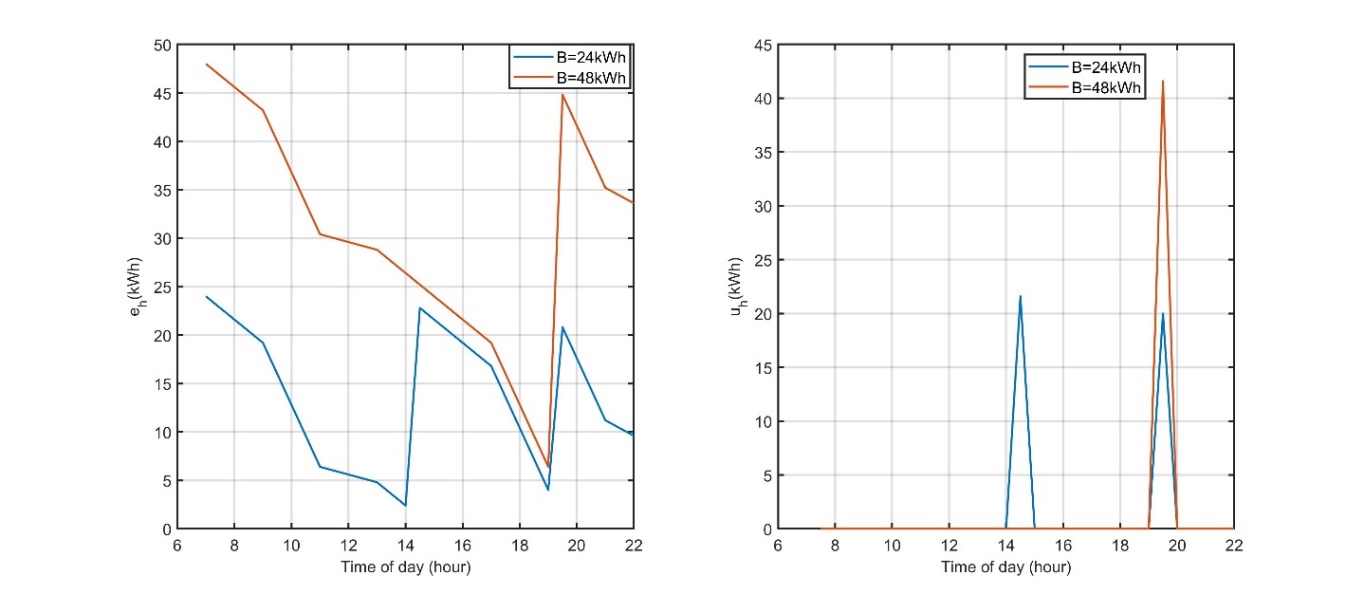


Figure A.2. Battery levels at the beginning of each epoch based on the on-need policy (Left). Recharged energy amounts in each epoch based on the on-need policy (Right).


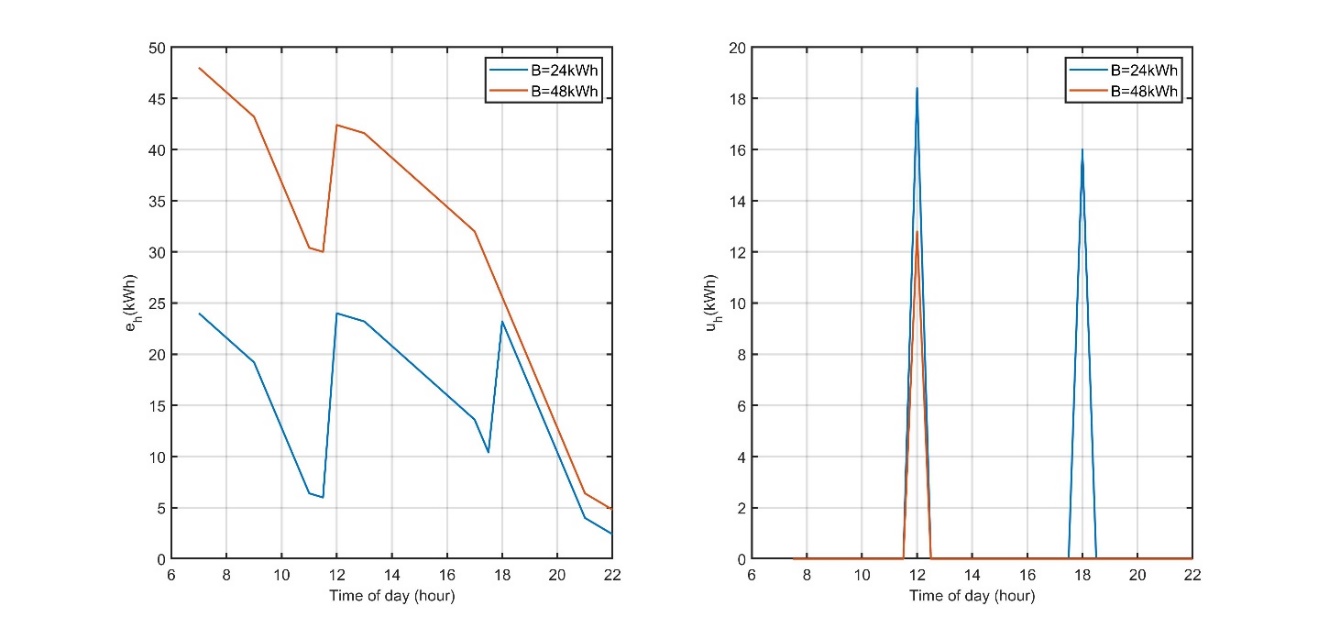


Figure A.3. Battery levels at the beginning of each epoch based on optimal battery recharge (Left). Recharged energy at each epoch based on optimal battery recharge (Right).

Table A.2. Total charging operation costs given different battery capacities.

| Battery capacity (kWh) | On-need policy  (euro) | Optimal charging schedule policy  (euro) | Saving |
| --- | --- | --- | --- |
| B=24 | 40.30 | 31.28 | –22.4% |
| B=48 | 36.04 | 8.71 | –75.8% |
