# Supplementary material for: Two-stage battery recharge scheduling and vehicle-charger assignment policy for dynamic electric dial-a-ride services
Source: PLoS One. 2021 May 20;16(5):e0251582. doi: 10.1371/journal.pone.0251582 (PMC8136635; doi:10.1371/journal.pone.0251582)
Supplement: S2 Appendix — (DOCX) [file pone.0251582.s002.docx]

## S2 Appendix. Illustrative example and computational study for optimal charging station assignment (P2).

The example is designed to illustrate the characteristics of the charging station assignment model. Consider 5 EVs with an identical battery capacity of 35.8 kWh and a full-charge driving range of 150 km, located at nodes 2, 5, 6, 7, and 9 with the same initial battery level of 20%, as shown in Figure B.1. The parameter settings for the illustrative example is shown in Table B.1. The charger power is identical at 40 kW for each charger. Vehicle speed is assumed to be 50 km/hour. The target energy levels to recharge for the vehicles, from left to right, are 80%, 40%, 50%, 80%, and 40%, respectively. There are a total of 4 chargers, of which 2 (A and B) are located at node 3 and the other two (C and D) at node 10. The available times for charger A and charger B are t=0 and t=40 min. (being occupied until t=40 min.), respectively. The available times for charger C and charger D are t=25 and t=20, respectively. The distance and travel time between any two adjacent nodes are 5 km and 6 min, respectively. As the number of vehicles is greater than that of chargers, the P2J problem is solved using the MATLAB intlinprog mixed-integer linear programming solver. The assignment results are shown in Figure B.1. The blue line represents the path that a vehicle takes to get to a charger and be charged at a higher State of Charge (SoC) level. The assignment results show that the total charging operation time is minimized with one-to-one vehicle–charger matches. Four vehicles are charged to its desired SoCs while the vehicle on the left side is not charged due to the charger availability limit (4 chargers vs. 5 vehicles) and its higher target charging level (80%). This demonstrates that when the number of vehicles to be charged is greater than that of chargers, the vehicles with a lower energy demand are prioritized for charging when all things are equal. This allows more vehicles to be available earlier to serve customers. Table B.2 shows the detailed result of the assignment model with the obtained value of the objective function Z*= 182.92. The arrival time, waiting time, and charging time of each vehicle are reported in Table B.2.


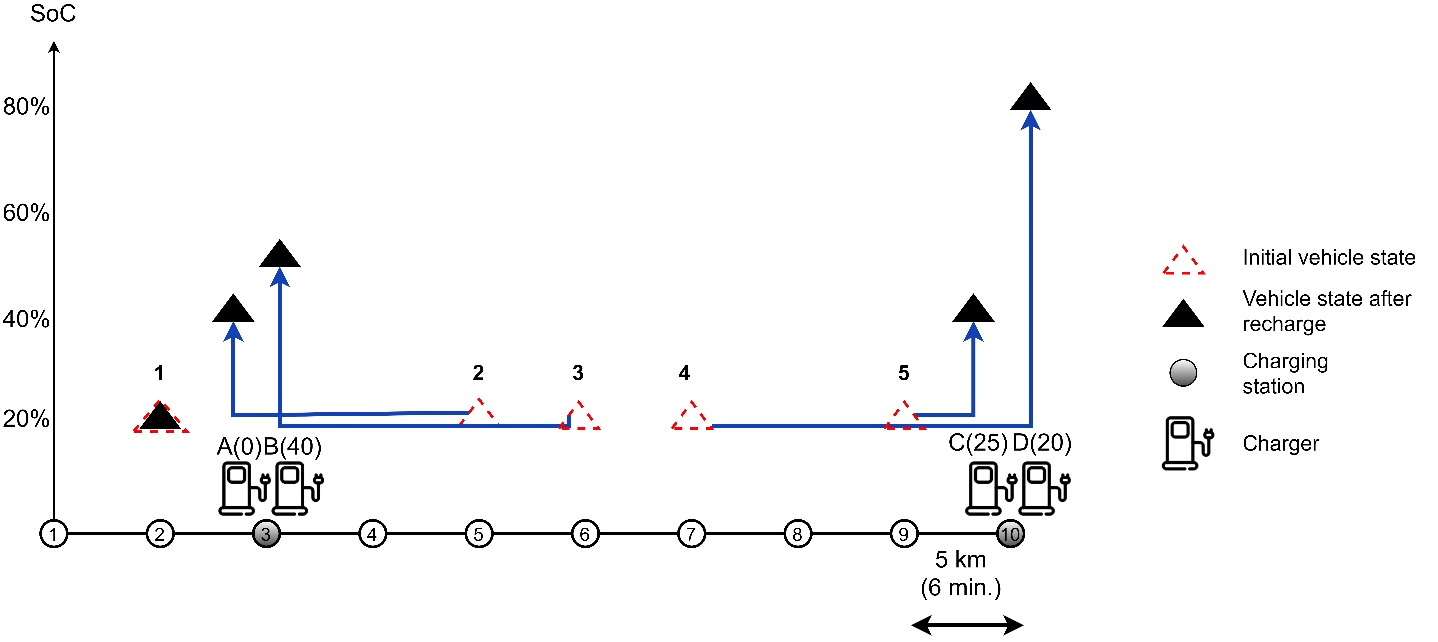


Figure B.1. Illustrative example of the charging station assignment model with 5 vehicles and 4 chargers (A,B,C, and D).

Table B.1. Parameter settings for the illustrative example.

| $B$ (battery capacity) | 35.8 (kWh) |
| --- | --- |
| $e_{min}$ | $0.1B$ |
| Driving range (full charge) | 150 (km) |
| $\theta_{1},\theta_{2}$ | 1 |
| $\varphi$ (charging rate) | 40 (kW/hour) |
| Location of chargers | (3,3,10,10) |
| Location of vehicles | (2,5,6,7,9) |
| $e_{i}$ | (0.2,0.2,0.2,0.2,0.2) *B (kWh) |
| Vehicle speed | 5/6 (km/min.) |
| $\varphi_{j},j=1,\ldots,4$ | 0.2386667 (kWh/km) |
| $t_{j}^{A},j=1,\ldots,4$ | (0,40,25,20) (min.) |
| $e_{i}^{*},i=1,\ldots,5$ | (0.8,0.4,0.5,0.8,0.4)*B |

Table B.2. Results of optimal charging assignment for the illustrative example.

| Vehicle | SoC (%of B) after recharge | Assigned charger | Arrival time | Waiting time | Charging time |
| --- | --- | --- | --- | --- | --- |
| 1 | 20 | - | - | - | - |
| 2 | 40 | A | 12.0 | 0.0 | 14.3 |
| 3 | 50 | B | 18.0 | 22.0 | 21.5 |
| 4 | 80 | D | 18.0 | 2.0 | 37.6 |
| 5 | 40 | C | 6.0 | 19.0 | 12.5 |
| Z* | 182.92 | | | | |

Remark: B is the battery capacity. Time is measured in minutes.

For larger problems, we generate two sets of test instances; each has 9 subsets of problems with different sizes of $\left| I \right|$ and $\left| J \right|$, and each subset has 3 randomly generated test instances. The first set of test instances is related to the P2 problem ($\left| I \right|\leq\left| J \right|$)$.$ The second set is related to the P2J problem ($\left| I \right|>\left| J \right|).$ Note that for the second set of instances, the number of chargers $\left| J \right|$ is randomly generated given $|J|<|I|.$ The locations of vehicles and chargers are randomly generated within a rectangular area within $(\left[ -50,50 \right]\times\left[ -50,50 \right])$. A vehicle’s initial energy level $e_{i}$, target energy level $e_{i}^{*}$ after recharge, and a chargers’ available time $t_{j}^{A}$ are randomly generated based on the parameters shown in Table B.3. The performance of the proposed heuristic is compared with the exact solution obtained by the MATLAB mixed-integer linear programming solver. In terms of the P2 problem size, the number of constraints in terms of Eqs. (11)–(16) is $\left| I \right|+\left| J \right|+4|I||J|$. The test instances are publicly available at <https://github.com/tym2021>.

Table B.3. Parameters for test instances generation for the Lagrangian relaxation algorithm.

| Variable | Value |
| --- | --- |
| *B* | 35.8 kWh |
| $e_{min}$ | $0.1B$ |
| $\vert I\vert$ | 10,20,30,40,50, 100,200,400, and 1000 |
| $\vert J\vert$ | 10,20,30,40,50, 100,200,400, and 1000 |
| Location of chargers/vehciles | rand$(\left[ -50,50 \right]\times\left[ -50,50 \right])$ |
| $e_{i}$ | rand(0.4,0.5)*B (kWh) |
| $e_{i}^{*}$ | rand (0.7,1) *B (kWh) |
| $t_{j}^{A}$ | rand (0,30) (min.) |
| $\theta_{1},\theta_{2}$ | 1 |
| $\varphi$ (charging rate) | 40 (kWh) |
| $\varphi_{j},j=1,\ldots,4$ | 0.2387 (kWh/km) |

The maximum number of iterations of the LR algorithm is set as 2000. Different values of the step size adjustment constant$0<\delta\leq2$ are tested and, finally, we pick 0.6 because it shows the best performance in most cases. The gap tolerance $\bar{\varepsilon}$ is set as 0.0001 for |I|<1000 and 0.005 otherwise. The computational results of the LR algorithm are shown in Table B.4. The results for the P2 problems are reported in the left column, while the right column reports the results for the P2J problems. The LR algorithm finds near-optimal solutions with an optimality gap of 0.5% in less than 3 minutes for the test instances of 1000 vehicles in the P2 and P2J problems. For the test instances of 200 vehicles, the optimality gap is around 0.16% for the P2 problem and 0.09% for the P2J problem, both found in around 30 seconds. However, the commercial exact solution solver cannot find solutions within 1 hour for |I|=50 and more. Our computational study shows that the proposed LR algorithm is suitable for large-scale real-time application for EV charging station assignment.

Table B.4. Computational results of the Lagrangian relaxation algorithm for the P2 and P2J problems.

|  | P2 | | | |  | P2J | | | | |
| --- | --- | --- | --- | --- | --- | --- | --- | --- | --- | --- |
| Prob. | $\vert I\vert$ | $\vert J\vert$ | Gap | CPU time (seconds) | | $\vert I\vert$ | $\vert J\vert$ | Gap | CPU time (seconds) | |
|  |  |  |  | LR | Exact |  | (avg.) |  | LR | Exact |
| 1 | 10 | 10 | 0 | 0.1 | 0.2 | 10 | 4.7 | 0.00% | 0.1 | 0.4 |
| 2 | 20 | 20 | 0.03% | 2.9 | 0.4 | 20 | 5.0 | 0.01% | 0.3 | 0.3 |
| 3 | 30 | 30 | 0.04% | 3.9 | 0.4 | 30 | 14.3 | 0.01% | 1.7 | 0.3 |
| 4 | 40 | 40 | 0.09% | 5.3 | 130.8 | 40 | 15.0 | 0.02% | 2.3 | 1.7 |
| 5 | 50 | 50 | 0.11% | 5.7 | NA | 50 | 37.3 | 0.03% | 4.5 | NA |
| 6 | 100 | 100 | 0.09% | 11.8 | NA | 100 | 51.3 | 0.03% | 12.5 | NA |
| 7 | 200 | 200 | 0.16% | 25.5 | NA | 200 | 117.0 | 0.09% | 36.5 | NA |
| 8 | 400 | 400 | 0.26% | 121.8 | NA | 400 | 104.3 | 0.06% | 115.4 | NA |
| 9 | 1000 | 1000 | 0.50% | 168.3 | NA | 1000 | 522.3 | 0.50% | 170.5 | NA |

Remark: 1. GAP =$\frac{Z_{UB}-Z_{LB}}{Z_{UB}}$. 2. NA means the exact solution cannot be found given a one-hour computation time.

3. The reported results are based on the average of three randomly generated test instances for each problem size.
