# Supplementary material for: Two-stage battery recharge scheduling and vehicle-charger assignment policy for dynamic electric dial-a-ride services
Source: PLoS One. 2021 May 20;16(5):e0251582. doi: 10.1371/journal.pone.0251582 (PMC8136635; doi:10.1371/journal.pone.0251582)
Supplement: S3 Appendix — (DOCX) [file pone.0251582.s003.docx]

## S3 Appendix. List of abbreviations of used terms.

| EV | Electric vehicles |
| --- | --- |
| FCFS | First-come-first-served minimum charging delay policy |
| LB | Lower-bound |
| LR | Lagrangian relaxation |
| NS | Need-based nearest charging station assignment policy |
| OCP | Proposed optimal charging scheduling and assignment policy |
| SAEV | Shared autonomous electric vehicles |
| TNC | Transport network company |
| UB | Upper bound |
